# Ovarian Response and Cumulative Live Birth Rate of Women Undergoing In-Vitro Fertilisation Who Had Discordant Anti-Mullerian Hormone and Antral Follicle Count Measurements: A Retrospective Study

**DOI:** 10.1371/journal.pone.0108493

**Published:** 2014-10-14

**Authors:** Hang Wun Raymond Li, Vivian Chi Yan Lee, Estella Yee Lan Lau, William Shu Biu Yeung, Pak Chung Ho, Ernest Hung Yu Ng

**Affiliations:** Centre of Assisted Reproduction and Embryology, Department of Obstetrics and Gynaecology, The University of Hong Kong, Queen Mary Hospital, Hong Kong; China Agricultural University, China

## Abstract

**Objective:**

To evaluate ovarian response and cumulative live birth rate of women undergoing in-vitro fertilization (IVF) treatment who had discordant baseline serum anti-Mullerian hormone (AMH) level and antral follicle count (AFC).

**Methods:**

This is a retrospective cohort study on 1,046 women undergoing the first IVF cycle in Queen Mary Hospital, Hong Kong. Subjects receiving standard IVF treatment with the GnRH agonist long protocol were classified according to their quartiles of baseline AMH and AFC measurements after GnRH agonist down-regulation and before commencing ovarian stimulation. The number of retrieved oocytes, ovarian sensitivity index (OSI) and cumulative live-birth rate for each classification category were compared.

**Results:**

Among our studied subjects, 32.2% were discordant in their AMH and AFC quartiles. Among them, those having higher AMH within the same AFC quartile had higher number of retrieved oocytes and cumulative live-birth rate. Subjects discordant in AMH and AFC had intermediate OSI which differed significantly compared to those concordant in AMH and AFC on either end. OSI of those discordant in AMH and AFC did not differ significantly whether either AMH or AFC quartile was higher than the other.

**Conclusions:**

When AMH and AFC are discordant, the ovarian responsiveness is intermediate between that when both are concordant on either end. Women having higher AMH within the same AFC quartile had higher number of retrieved oocytes and cumulative live-birth rate.

## Introduction

Ovarian response markers, such as serum follicle stimulating hormone (FSH) concentration, antral follicle count (AFC) and serum anti-Mullerian hormone (AMH) concentration are often employed in in vitro fertilisation (IVF) programmes to predict ovarian response to gonadotrophin stimulation [1], [2]. Systematic reviews showed that among the common ovarian response markers, AFC and AMH had the best and comparable performance in predicting both poor and excessive ovarian responses [3], [4], [5]. Nonetheless, the prediction of pregnancy outcomes by these markers is poor [4], [6], [7]. Hence, the clinical applications of these markers are mainly in prognostic counseling as well as in individualization of the stimulation regimen.

AMH, also known as Mullerian-inhibiting substance, is a dimeric glycoprotein hormone which is a member of the transforming growth factor-beta family. In adult women, AMH is exclusively produced by granulosa cells of preantral and small antral follicles, and has been shown to have excellent correlation with the primordial follicle pool [8]. Hence, AMH may reflect ovarian reserve as well.

AMH and AFC have been shown to have a very good correlation with each other, although AFC may be more machine- and operator-dependent and could have higher inter-operator variability compared to AMH. Yet, discordance between AMH and AFC does occur in some individual women. To our knowledge, there have been no reported data on the implications of ovarian response and success rate in women undergoing IVF treatment who had discordant AMH and AFC. On the other hand, studies have revealed considerably high frequency of discordance between AMH and FSH in women undergoing IVF [9], [10]. Therefore, we conducted this retrospective analysis to evaluate the discordance frequency between AMH and AFC in women undergoing IVF treatment, and its implication on the ovarian response and the cumulative live birth outcome.

## Materials and Methods

### Subject selection

This retrospective review included all first IVF cycles carried out between January 2007 and December 2009 at the Centre of Assisted Reproduction and Embryology, The University of Hong Kong – Queen Mary Hospital, Hong Kong, excluding those involving the use of donor oocytes, blastocyst transfer and/or pre-implantation genetic diagnosis. Only the first treatment cycle of each subject in our Centre was included for this study, regardless of whether the subjects had previous IVF treatment elsewhere. Those with blastocyst transfer and/or pre-implantation genetic diagnosis were not included in this study because the extended embryo culture would have altered the total number of replaceable embryos and hence the cumulative live birth outcome. A similar cohort of subjects has been used in another study recently published by our group [7], to which details can be referred. Only subjects treated on the long GnRH agonist protocol were selected for the current study. Ethics approval was obtained from the Institutional Review Board of the University of Hong Kong/Hospital Authority Hong Kong West Cluster for this retrospective study to be carried out using existing patient data in an anonymous manner, waiving the requirement for written consent from individual patients.

### Stimulation cycle, AFC and AMH determination, and frozen-thawed embryo transfer

Details of the stimulation cycle have been reported previously [7]. In the long GnRH agonist protocol, the subject received buserelin (Suprecur, Hoechst, Frankfurt, Germany) nasal spray starting from the mid-luteal phase of the cycle preceding the stimulation cycle. In the early follicular phase of the treatment cycle after return of period, transvaginal ultrasound scan was performed using a 5 MHz vaginal probe (Voluson 730, GE Healthcare, Wisconsin, USA) to count the total number of antral follicles ranging from 2 to 9 mm. All ultrasound scans were performed by reproductive medicine specialists who had at least 7 years of experience in gynaecological ultrasound and had been trained under the same supervisor. Blood was taken for routine estradiol measurement, and the residual serum samples were archived at −20°C. For the current study, these stored serum samples were retrieved for AMH measurement using the AMH Gen II ELISA kit (Beckman Coulter, Webster, TX, USA; catalogue number A79765). The assay kit has a sensitivity of 0.08 ng/ml, and intra- and inter-assay coefficients of variation of less than 5.4 and 5.6%.

After confirming a basal serum estradiol level, the subjects received human menopausal gonadotrophin (HMG) or recombinant FSH for ovarian stimulation. They continued the buserelin nasal spray till human chorionic gonadotrophin (hCG) trigger. The initial dose of gonadotrophin stimulation was decided based on the baseline AFC (AFC≥15: 150 IU per day; AFC between 6 and 14: 300 IU for the first two days followed by 150 IU daily; AFC≤5: 300–450 IU for the first two days followed by 225 IU daily). hCG (Pregnyl 5000 or 10000 units or Ovidrel 250 µg) was given to trigger final oocyte maturation when there were at least 3 follicles reaching 16 mm or above in mean diameter, with the leading one reaching 18 mm. Transvaginal ultrasound-guided oocyte retrieval was carried out 36 hours later. In-vitro fertilisation was performed either by conventional insemination or intracytoplasmic sperm injection (ICSI) depending on semen parameters. Embryo replacement was carried out two days after fertilization. A maximum of two embryos were allowed per transfer.

Surplus embryos of grades 1 to 4 [11], [12] were cryopreserved on the day of the embryo transfer. Frozen-thawed embryo transfers were carried out in natural cycles for ovulatory women, and in clomiphene-induced cycles or hormone replacement cycles for anovulatory women. Details of the embryo cryopreservation and frozen-thawed embryo transfer protocols have been previously described [12].

### Grouping of subjects according to upper and lower quartiles of AMH and AFC

Using data of all the 1,152 women undergoing the first IVF cycle during the study period, the 25^th^ percentile and 75^th^ percentile values of serum AMH concentration were 1.4 ng/ml and 5.3 ng/ml respectively, whereas those of AFC were 6 and 14 respectively. Our subjects were then grouped according to whether their AMH and AFC values were in the lower quartile, upper quartile or in between (Table 1).

**Table 1:** Grouping of subjects according to the quartile of serum AMH and AFC values.

Serum AMH (ng/ml)	Antral follicle count (AFC)			
	<6	6–14	>14	Total
**<1.4**	**Group 1**	Group 2	Group 3	(207)
	**(128)**	(78)	(1)	
**1.4–5.3**	Group 4	**Group 5**	Group 6	(555)
	(70)	**(395)**	(90)	
**>5.3**	Group 7	Group 8	Group 9	(284)
	(4)	(94)	(186)	
**Total**	(202)	(567)	(277)	(1046)

The number of subjects in each group is indicated in brackets.

### Statistical analysis

The primary outcome measures were the number of oocytes retrieved, ovarian sensitivity index (OSI =  number of retrieved oocytes per 1,000 IU of gonadotrophin (FSH) administered) [13], and the cumulative live birth in the fresh plus all frozen-thawed embryo transfers following the same index stimulation cycle. Non-normally distributed continuous variables were expressed as median (interquartile range) unless otherwise stated. Continuous and categorical variables were compared between groups using Mann-Whitney test or Kruskal-Wallis test with Conover post-hoc analysis, and Fisher's Exact test respectively. Statistical analysis was carried out using the IBM SPSS Statistics (Version 20, IBM Corporation, U.S.A.) and MedCalc (Version 12.5, MedCalc Software, Belgium). The two-tailed value of P<0.05 was considered statistically significant.

## Results

There were 1,152 women undergoing the first IVF treatment cycle during the study period, out of which we studied on 1,046 subjects who fulfilled the inclusion criteria and being treated on the long GnRH agonist protocol. The clinical and demographic parameters of the included subjects are listed in Table S1.

### Discordance between AMH and AFC

As shown in Table 1, subjects in Groups 1, 5 and 9, i.e. 709 out of 1046 (67.8%), were concordant in AMH and AFC. This means that the rest 337 subjects (32.2%) showed discordance between AMH and AFC. There was no significant difference (p>0.05) in body mass index among the groups.

### Number of retrieved oocytes as stratified by subject grouping

Comparing the groups within the same AFC category, i.e. 1 vs 4, 2 vs 5 vs 8 and 6 vs 9, the groups with higher AMH levels had significantly higher numbers of oocytes retrieved (Table 2).

**Table 2:** Number of retrieved oocytes in different subject groups classified according to the quartile of serum AMH and AFC.

Serum AMH (ng/ml)	Antral follicle count (AFC)		
	<6	6–14	>14
**<1.4**	**Group 1**	Group 2	Group 3
	**4 (3–7)^a^**	6 (4–8)^b^	15∧
**1.4–5.3**	Group 4	**Group 5**	Group 6
	8 (6–10)^a^	**9 (6–11)^b,c^**	10 (7–15)^d^
**>5.3**	Group 7	Group 8	**Group 9**
	12.5 (10–16)	12 (9–16)^c^	**14 (11–18)^d^**

Values are expressed in median (interquartile range).

a,b,c,d p<0.0001 (statistically significant. Mann-Whitney U test).

∧Only 1 case.

### Cumulative live birth rate as stratified by subject grouping

Group 4 had a significantly higher cumulative live birth rate compared to group 1. Likewise, the cumulative live birth rate was significantly higher in group 8 than in group 5, which was in turn higher than in group 2. Similarly, group 9 had significantly higher cumulative live birth rate compared to group 6 (Table 3).

**Table 3:** Cumulative live birth rate in different subject groups classified according to the quartile of serum AMH and AFC.

Serum AMH (ng/ml)	Antral follicle count (AFC)		
	<6	6–14	>14
**<1.4**	**Group 1**	Group 2	Group 3
	**40/112 (35.7%)** a	24/61 (39.3%)^b^	1/1 (100.0%)
**1.4–5.3**	Group 4	**Group 5**	Group 6
	36/65 (55.4%)^a^	**206/372 (55.4%)** b **^,^** c	48/85 (56.5%)^d^
**>5.3**	Group 7	Group 8	**Group 9**
	1/1 (100.0%)	63/86 (73.3%)^c^	**114/164 (69.5%)** d

Values are expressed in median (interquartile range).

a p = 0.012 (statistically significant, Fisher's Exact test).

b p = 0.026 (statistically significant, Fisher's Exact test).

c p = 0.002 (statistically significant, Fisher's Exact test).

d p = 0.0497 (statistically significant, Fisher's Exact test).

### OSI as stratified by subject grouping

Among groups 1, 2, 4 and 5, the OSI of groups 2 and 4 (i.e. the discordant groups) was significantly lower than that of group 1 and higher than that of group 5 (p<0.05 for both comparisons). Likewise, the OSI of groups 6 and 8 (i.e. the discordant groups) was significantly lower than that of group 5 but higher than that of group 9 (p<0.05 for both comparisons). There was no significant difference in OSI between groups 2 and 4, and between groups 6 and 8 (p>0.05) (Table 4).

**Table 4:** Ovarian sensitivity index in different subject groups classified according to the quartile of serum AMH and AFC.

Serum AMH (ng/ml)	Antral follicle count (AFC)		
	<6	6–14	>14
**<1.4**	**Group 1**	Group 2	Group 3
	**1.3 (0.8–2.1)^a,b,c^**	2.2. (1.4–3.3)^a,d^	10.0∧
**1.4–5.3**	Group 4	**Group 5**	Group 6
	2.4 (2.1–3.6)^b,e^	**3.8 (2.4–5.5)^c,d,e,f,g,h^**	6.5 (3.7–8.2)^f,i^
**>5.3**	Group 7	Group 8	**Group 9**
	5.8 (4.3–6.5)	6.5 (4.7–9.3)^g,j^	**8.8 (5.9–12.6)^h,i,j^**

Values are expressed in median (interquartile range).

∧Only 1 case.

Comparison I (between Groups 1, 2, 4 and 5):

a,b,c,d,e p<0.05 (Kruskal-Wallis test with Conover post-hoc analysis).

Comparison II (between Groups 5, 6, 8 and 9):

f,g,h,i,j p<0.05 (Kruskal-Wallis test with Conover post-hoc analysis).

## Discussion

Many studies have consistently reported that AMH and AFC were among the best ovarian response markers for prediction of ovarian response in IVF treatment, and that their performance was probably comparable [4], [5], [7]. Strong significant correlations between AMH and AFC have also been demonstrated. This is explainable by the fact that AMH is produced from granulosa cells of small antral follicles. However, to our knowledge, there has been no report on the incidence or implication of discordance in AMH and AFC in these subjects. On the other hand, there have been some reports on discordance between AMH and FSH, the more conventional ovarian reserve marker, in women undergoing IVF [9], [10]. To decide what best to be done when the two markers give discordant results is a real practical issue.

We studied ovarian response in terms of the number of retrieved oocytes as well as OSI. OSI, which refers to the number of oocytes retrieved per 1000 IU gonadotrophin administered, is a measure of ovarian responsiveness. A strong correlation of OSI with the number of retrieved oocytes and other measures of ovarian response has been demonstrated by our group [13]. The use of this ratio in our study eliminates the confounding effect of the different initial doses of gonadotrophin being used across the different subject groups based on baseline AFC; this allows more appropriate comparison of ovarian response between groups.

Our results showed that a significant proportion, amounting to more than 30%, of subjects undergoing IVF treatment were discordant in AMH and AFC as classified by their quartiles. The discordant groups with low to normal ovarian reserve had ovarian responsiveness intermediate between those with concordantly low and concordantly normal AMH and AFC. Likewise the discordant groups with normal to high ovarian reserve had ovarian responsiveness intermediate between those with concordantly normal and concordantly high AMH and AFC. Within each AFC category, those with higher AMH had significantly higher number of oocytes retrieved and OSI. This finding was compatible with our current understanding from most available studies. As AMH is chiefly secreted from ovarian granulosa cells in the adult women, it serves as an index of the follicular pool size [8] which directly determines ovarian response.

We also studied the implication on the cumulative live birth rate when subjects in the same AFC category, hence subjected to the same stimulation regimen, were in different AMH categories. In such circumstances, those with discordantly higher AMH category generally had significantly higher cumulative live birth rates. Since the cumulative live birth is mainly determined by the total number of transferrable embryos from the same index stimulation cycle [7], which is in turn dependent on the number of oocytes retrieved, it is not surprising that the implications of the discordance between AMH and AFC on the cumulative live birth mirrored that on ovarian responsiveness on the whole.

Likewise, when comparing groups within the same AMH category, those having higher AFC did have higher OSI, as shown in Table 4. However, we could not analyse the number of oocytes and cumulative live birth rate in this manner as in our protocol there was inherent variation of stimulation dose based on AFC.

The reason of the discordance between AMH and AFC is not absolutely certain. It may be due to the fact that the serum AMH level is not exactly reflecting the population of antral follicles counted in our AFC measurement, i.e. those between 2 to 9 mm in size. It has been suggested that the peak production of AMH occurs in small antral follicles up to 8 mm [14], beyond which it starts to drop. Another study showed that AMH concentration in follicular fluid dropped with follicular size from 3 mm to 9 mm, and reached negligible level when the follicle grew beyond 10 mm [15]. In other words, what we counted in the AFC measurement might not all be producing the same amount of AMH concurrently. On the other hand, not all the preantral and small antral follicles contributing to the AMH measured would subsequently develop to maturity leading to oocyte being yielded. Moreover, there could be intra- and inter-operator variability of AFC measurement, as well as intra- and inter-assay variability of AMH measurement, which may contribute to discordance in some cases.

In this study, we classified the AMH and AFC measurements as low and high based on their respective lower and upper quartiles. The cut-offs of 6 and 14 for AFC actually were compatible with those suggested by previous studies for poor and excessive responders [16], [17]. The cut-offs for AMH adopted in this study was slightly higher numerically compared to most previous studies [16], [17], which actually varied across a wide range mainly because of different AMH assay methods used. In this study, AMH was measured by the Beckman-Coulter Gen II ELISA kit, which was introduced relatively recently. This is the current one in the market, but was not what most previous reports were based on.

The current study only included those treated on the long GnRH agonist protocol so as to avoid the potential confounding effect of different protocols on the outcome measures. As over 90% of our subjects were treated on the long GnRH agonist protocol during the studied period, this gave a fair representation of the overall patient cohort. Repeating the analyses after inclusion of those treated on the GnRH antagonist protocol gave the same conclusion (data not shown). Only the first treatment cycle of each subject in our centre was included in this analysis so as to avoid bias arising from over-representation of subjects receiving multiple treatment cycles during the study period. Although some subjects had received previous IVF treatment elsewhere, this should not have affected the conclusion drawn in this study. The AMH and AFC were measured at commencement of ovarian stimulation after pituitary down-regulation. Our group has previously shown that AFC is not significantly altered by short-term GnRH agonist treatment for pituitary down-regulation [18]. Our own cohort also showed that GnRH agonist down-regulation did not significantly alter serum AMH measurement compared to pre-down-regulation level (unpublished data); it has also been reported that AMH measurement at the start of ovarian stimulation in IVF patients down-regulated with GnRH agonist still had good prediction of ovarian response [19].

In all, our results suggested that when AMH and AFC are discordant, those having higher AMH within the same AFC quartile had higher number of retrieved oocytes and cumulative live birth rate, and the ovarian responsiveness is intermediate between that when both are concordant on either end. Both AMH and AFC would be recommended to be utilized for individualization of stimulation regimen; when the AMH and AFC fall into discordant categories, it would be sensible to adopt an intermediate dose of gonadotrophin as compared to those with concordant AMH and AFC categories on either end. Such theoretical consideration, however, is yet to be proven in further clinical trials which are keenly awaited.

## Supporting Information

Table S1 **Clinical and demographic parameters of the subjects included in this study.**
(DOCX)
