# Supplementary material for: Ovarian Response and Cumulative Live Birth Rate of Women Undergoing In-Vitro Fertilisation Who Had Discordant Anti-Mullerian Hormone and Antral Follicle Count Measurements: A Retrospective Study
Source: PLoS One. 2014 Oct 14;9(10):e108493. doi: 10.1371/journal.pone.0108493 (PMC4196774; doi:10.1371/journal.pone.0108493)
Supplement: Table S1 — Clinical and demographic parameters of the subjects included in this study. (DOCX) [file pone.0108493.s001.docx]

**Supplemental Table S1.** Clinical and demographic parameters of the subjects included in this study.

| **Parameters** | **Antral follicle count (AFC)** | | | |  |
| --- | --- | --- | --- | --- | --- |
|  | **<6** | **6 – 14** | **>14** | **P value** | **Total** |
| N | 202 | 567 | 277 |  | 1046 |
| Age (years) | 37  (34 – 38) | 36  (33 – 38) | 34  (31 – 36) | <0.001* | 35  (33 – 38) |
| Body mass index (kg/m^2^) | 21.2  (19.4 – 23.3) | 21.1  (19.6 – 22.9) | 21.4  (19.7 – 23.5) | 0.345 | 21.2  (19.6 – 23.1) |
| Duration of subfertility (years) | 4 (3 – 6) | 4 (3 – 6) | 4 (3 – 6) | 0.651 | 4 (3 – 6) |
| Cause of subfertility  Tubal  Endometriosis  Male  Unexplained  Mixed  Others | 31 (15.3%)  7 (3.5%)  118 (58.4%)  7 (3.5%)  39 (19.3%)  0 (0.0%) | 71 (12.5%)  30 (5.3%)  336 (59.3%)  31 (5.5%)  96 (16.9%)  3 (0.5%) | 40 (14.4%)  6 (2.2%)  180 (65.0%)  12 (4.3%)  32 (11.6%)  7 (2.5%) | 0.009* | 142 (13.6%)  43 (4.1%)  634 (60.6%)  50 (4.8%)  167 (16.0%)  10 (1.0%) |
| Type of subfertility  Primary  Secondary | 126 (62.4%)  76 (37.6%) | 383 (67.5%)  184 (32.5%) | 191 (69.0%)  86 (31.0%) | 0.286 | 700 (66.9%)  346 (33.1%) |
| Smoking  Yes  No | 24 (11.9%)  178 (88.1%) | 51 (9.0%)  516 (91.0% | 18 (6.5%)  259 (93.5%) | 0.123 | 93 (8.9%)  953 (91.1%) |
